# Two-thirds of pregnant women attending antenatal care clinic at the University of Gondar Hospital are found with subclinical iodine deficiency, 2017

**DOI:** 10.1186/s13104-018-3829-0

**Published:** 2018-10-17

**Authors:** Wubet Worku Takele, Mekuriaw Alemayehu, Terefe Derso, Amare Tariku

**Affiliations:** 1 Department of Community Health Nursing, School of Nursing College of Medicine and Health Sciences, University of Gondar, Gondar, Ethiopia; 2 Department of Environmental Health, Institute of Public Health, College of Medicine and Health Sciences, University of Gondar, Gondar, Ethiopia; 3 Department of Human Nutrition, Institute of Public Health, College of Medicine and Health Sciences, University of Gondar, Gondar, Ethiopia

**Keywords:** Pregnant women, Subclinical iodine deficiency, Ethiopia

## Abstract

**Objective:**

This study was aimed at determining the magnitude of prenatal iodine deficiency and its determinants among women attending antenatal care clinic at the University of Gondar Specialized Referral Hospital, Northwest Ethiopia. A cross-sectional study was conducted from March 13 to April 25/2017. Precisely, 378 pregnant women were included in the study selected via systematic random sampling technique. Urinary Iodine concentration was determined through spectrophotometer using Sandell-Kolthoff reaction. Iodine deficiency was defined as women having urinary iodine concentration of < 150 µg/L. Moreover, stool examination was done.

**Results:**

Subclinical iodine deficiency among pregnant women was 60.5% (95% CI 55%, 65.5%). The Median iodine concentration was 137 μg/L (IQR 80 μg/L). Being governmental employee [AOR = 0.42 (95% CI 0.1 = 20, 0.87)], cabbage consumption of twice or more times per week [AOR = 2.35 (95% CI 1.44, 3.82)], not consuming maize in the last 1 week [AOR = 0.29 (95% CI 0.18, 0.48)], poor household wealth status [AOR = 2.7 (95% CI 1.24, 5.89)], and second trimester of pregnancy [AOR = 2.43 (95% CI 1.37, 4.32)] were significantly associated with iodine deficiency. Prenatal iodine deficiency was high, which deemed a mild public Health problem. Therefore, improving household income, and nutrition education to minimize maize and cabbage consumption are recommended.

## Introduction

Iodine is an essential trace mineral, which is vital for the synthesis of Thyroid Hormones, as triiodothyronine (T3), and thyroxine (T4). These hormones are crucial for healthy growth and the development of the brain [1, 2]. In consonance with the World Health Organization (WHO), prenatal iodine deficiency (ID) is defined as urinary iodine concentration of < 150 µg/L [3]. To eliminate ID at the country level, the salt iodization Council of Ministers enacted regulation No. 204/12011 on the implementation of universal iodized salt utilization [4]. ID in Africa, Nigeria [5], Niger [6], and Ethiopia [7] bear 100%, 61.67, and 88.9% of the burden, respectively.

Prenatal ID exposes the newborn to long-term irreversible sequelae of brain damage following poor migration and myelination of neurons [8, 9]. Consequently, it compromises about 12–13.5 points of the newborn’s Intelligence Quotient (IQ) [10], which result in poor educational outcomes [11, 12]. Moreover, low birth weight and poor linear growth are another bad consequences of prenatal ID [13, 14].

People with dietary habits of cassava, cabbage, sorghum, and millet as staple foods are iodine-deficient [15]. Besides, poor storage of salt, utilization of non-packed salt, and lack of knowledge towards iodized salt are extra factors of ID [16]. Iodized salt utilization in Ethiopia is far lower (23.3%) than [17, 18] WHO’s recommendation (90%) [19].

Even though ID is one of the grave concerns in Ethiopia, little is known about the burden among pregnant women. Therefore, this study was aimed at investigating prenatal ID and associated factors among women attending the antenatal clinic at the University of Gondar Referral Hospital.

## Main text

### Methods

#### The study area, design, and population

This facility-based cross-sectional study was conducted from March 13 to April, 25/2017 among pregnant women attending ANC at the University of Gondar Referral Hospital. Participants with an established hypertensive disorder as their dietary salt restriction were excluded.

#### Sample size, Sampling technique, and procedure

The sample size was calculated considering the statistical assumptions for the prevalence of ID as 61.4% [20], 95% level of confidence, 5% margin of error, and 10% non-response rate, which yielded a sample size of 403. The systematic random sampling technique was employed. The average number of pregnant women attending the ANC clinic in the preceding one and half months was estimated to be 1545. Accordingly, a sampling fraction of 4 (Kth = N/n) was calculated. Following the selection of the first participant using the lottery method, every fourth woman of the initial participant was included. Nevertheless, the next participants were considered provided that the selected ones did not fulfill the inclusion criteria.

#### Data collection procedures and tools

The data were collected using a face-to-face interviewer-administered questionnaire. It comprised of socio-demographic characteristics, obstetric, and dietary practices.

Five-milliliter single-spot urine was collected using a clean plastic neck-tube with a tightly screw-cap labeled with participant’s identification number. The collected urine sample was stored in a cold-box for ease of transportation and kept below − 20 °C in the refrigerator until analysis. Furthermore, the stool was collected using a clean stool cup and processed within 30 min.

#### Assessment of urinary iodine concentration and Goiter

According to the WHO, prenatal ID is defined as women having urinary iodine concentration of < 150 µg/L. Maternal goiter status was diagnosed by physical examination of the neck. The severity of goiter was categorized as Grade 0 when there was no palpable and visible goiter; Grade 1, palpable but not visible, while the neck was in normal position, whereas Grade 2 was described as visible and palpable.

#### Maternal nutritional status and dietary diversity measurement

Maternal nutritional status was determined by Mid Upper Arm Circumference (MUAC). Accordingly, the participant was considered as underweight if her MUAC measurement was < 23 cm.

The minimum dietary diversity was measured using a 24-h-recall method to interview participants to report the food item consumed in the previous 24 h prior to the date of data collection. Accordingly, minimum dietary diversity was defined as when a woman consumed five and above food items [21].

#### Laboratory analysis

Urine iodine level was measured by the Sandell-Kolthoff reaction method using ammonium persulfate as a digestion recommended by WHO/UNICEF/International Council for Control of Iodine Deficiency Disorder (ICCIDD) [22]. Then, the sample was analyzed by the *Varian Cary, 50 UV*–*Vis spectrophotometer*-*Agilent, Malaysia)* machine. Likewise, stool wet mount test was prepared using saline and examined microscopically to detect intestinal Helminthes and protozoal Parasitosis.

#### Data analysis

The collected data were analyzed by STATA Version 14. Frequencies, percentages, and a measure of central tendencies with the appropriate measure of dispersion were used. Data were presented using tables, and text. The binary logistic regression was fitted to identify factors associated with the ID. All variables with p-values of < 0.2 were entered into the multi-variable analysis model to control the possible effects of a confounder. According to the variance inflation factor (VIF), there was no multicollinearity problem. Finally, variables independently associated with ID were identified on the basis of the Adjusted Odds Ratio (AOR) with a 95% CI and less than 0.05 *p* value.

#### Ethical approval

The ethical clearance was obtained from the Institutional Review Board of the University of Gondar. Written informed consent was obtained from each study participants. The study participant’s confidentiality was maintained by avoiding possible identifiers, such as the name of the patient. During data collection time any woman who was with medical problem findings, such as malnutrition, nutrition education was provided. Likewise, women with intestinal parasitic infection were treated by appropriate medications. Women with grade one and two goiters were linked to the surgical department.

### Results

#### Sociodemographic characteristics of pregnant women

About 403 participants were included in the study making a response rate of 94%. The mean (± SD) age of the participants was 26.3 (± 5.8) years. More than half of the participants (55%) were in the age range of 25–34 years (Table 1).

**Table 1:** Sociodemographic characteristics of pregnant women attending ANC service at the University of Gondar Referral Hospital, Northwest Ethiopia, 2017 (n = 378)

Characteristics	Frequency	Percentage
Age		
15–24	126	33.3
25–34	208	55
35–49	44	11.6
Residence		
Rural	66	17.5
Urban	312	82.5
Religion		
Orthodox	340	89.9
Muslim	33	8.7
Others	5	1.4
Ethnicity		
Amhara	356	94.2
Tigre	22	5.8
Women’s Educational status		
Unable to read and write	22	5.8
Primary	129	34.1
College and above	227	60.1
Women’s occupation		
Governmental worker	99	26.2
Merchant	54	14.3
Housewife	200	52.9
Daily laborer	25	6.6
Husband educational status		
Unable to read and write	85	25.1
Primary	74	22.3
College and above	188	52.6
Husband employment		
Governmental worker	144	38.1
Merchant	78	23.3
Unemployed	54	16.9
Farmer	30	10.8
Daily laborer	41	10.8
Water source		
Tap water	359	96
Spring/river	19	4
Toilet		
Flush to piped sewer	126	33.3
Flush to the septic tank	28	7.4
Ventilate improved Pit (VIP) latrine	86	22.7
VIP without slab	88	23.2
Open field	50	13.2

#### Health, the dietary and nutritional status of the pregnant women

Approximately one-fifth (19.6%) of the participants lied in the category of underweight. One in every twenty women were infected with hookworm.

Nearly one-third (32%) of women consumed a diversified diet. About 61.4% of the participants consumed cabbage twice or more in a week. Besides, about 96%, 92.3%, and 97.9% of women never consumed sweet potato, soya bean, and fish in the previous 1 week, respectively.

#### Salt utilization characteristics

The vast majority (88.1%) of pregnant women habitually consume packed salt. Most of the participants (96%), and (98.4%) they hardly expose the salt to sunlight and wash salt to avoid impurities, respectively (Table 2).

**Table 2:** Salt utilization characteristics of pregnant women attending ANC service at the University of Gondar referral hospital, Northwest Ethiopia, 2017 (n = 378)

Characteristics	Frequency	Percentage
Types of salt utilization		
Packed	333	88.1
Non packed	45	11.9
Exposure to sunlight/fire		
Never	363	96.0
Sometimes	15	4.0
Salt storage		
Dry	212	56.1
Moist	166	43.9
Do you wash the salt		
Never	372	98.4
Sometimes	6	1.6
Salt containing container		
Open	35	9.3
Closed	343	90.7
The timing of salt added to the food		
At the beginning	6	1.6
At the middle	15	4.0
At the end	357	94.4
Salt storage duration (months)		
≤ 2	354	93.7
≥ 3	24	6.3

#### Pregnant women attitude and knowledge towards iodized salt utilization and its importance

More than half (55.8%) of pregnant women had a favorable attitude to iodized salt utilization and ID, whereas 50.1% of study participants have adequate knowledge.

#### Iodine deficiency

Prenatal sub-clinical ID was 60.5% (95% CI 55.6, 65.5) with the Median Urinary Iodine Concentration (MUIC) of 137 μg/L (IQR 80 μg/L) (< 20 μg/L). In addition, the goiter rate was reported to be 38%.

#### Factors associated with ID

The odds of ID were decreased by 58% (AOR = 0.42 (95% CI (0.20, 0.87)) among pregnant women whose husbands were governmental employees as compared to whose husbands who were unemployed. The likelihood of ID was higher by 2.4 folds among women in the second trimester than the third trimester, 2.43 (AOR = 2.43 (95% CI 1.37, 4.32). The odds of ID were higher among participants who consumed cabbage (AOR = 2.35 (95% CI 1.44, 3.82), and women lived in poor household income (AOR = 2.7 (95% CI 1.24, 5.89). A decreased odds of ID were detected among women who never consumed maize in the previous 1 week compared to their counterparts (AOR = 0.29 (95% CI 0.18, 0.48) (Table 3).

**Table 3:** Factors associated with iodine deficiency among pregnant women attending ANC service at University of Gondar Referral Hospital, Northwest Ethiopia, 2017 (n = 378)

Variables	Urine iodine status		Crude odds ratio (COR)	Adjusted odds ratio (AOR)
	Deficient	Not deficient		
Husband employment				
Governmental	81	63	0.54 (0.28, 1.01)	0.42 (0.20, 0.87)**
Merchant	54	34	0.67 (0.33, 1.33)	0.57 (0.26, 1.26)
Farmer	24	17	0.59 (0.26, 1.35)	0.58 (0.22, 1.56)
Daily laborer	25	16	0.65 (0.28, 1.5)	0.72 (0.28, 1.83)
Unemployed	45	19	1.00	1.00
Trimester				
First trimester	35	21	1.36 (0.67, 2.62)	1.3 (0.65, 2.72)
Second trimester	77	27	2.4 (1.4, 4.11)	2.43 (1.37, 4.32)**
Third trimester	117	101	1.00	1.00
Gravidity				
Primi-gravida	158	29	1.54 (0.77, 3.09)	
Multigravida	71	101	1.00	
Parity				
Nulliparous	97	79	1.00	
Para one	72	33	1.50 (0.73, 3.09)	
Multiparous	60	37	1.05 (0.48, 2.30)	
Cabbage consumption				
≤ 1/week	73	73	1.00	1.00
≥ 2 times/week	156	76	2.05 (1.34, 3.13)	2.35 (1.44, 3.82)***
Sorghum consumption in the previous 1 week				
Yes	89	46	1.42 (0.9, 2.2)	*
No	140	103	1.00	1.00
Maize consumption in the previous 1 week				
Yes	129	45	1.00	1.00
No	100	104	0.33 (0.21, 0.51)	0.29 (0.18, 0.48)***
Toilet				
Flush to piped sewer	72	54	1.00	
Flush to septic tank	20	8	1.8 (0.76, 4.57)	
Ventilate improved Pit (VIP) latrine	60	26	1.7 (0.96, 3.09)	
VIP without slab	45	43	0.7 (0.45, 1.35)	
Open field	32	18	1.3 (0.67, 2.62)	
Wealth index				
Richest	35	40	1.00	1.00
Rich	46	30	1.(0.91, 3.34)	1.56 (0.76, 3.22)
Middle	47	26	2 (1.06, 3.9)	1.88 (0.88, 3.97)
Poorer	52	23	2.5 (1.32, 5.04)	2.7 (1.24, 5.89)**
Poorest	49	30	1.8 (0.98, 3.540	1.38 (0.65, 2.91)

1.00 reference category

*** p value < 0.001

** p value < 0.01

* p-value < 0.05

### Discussion

Prenatal ID is the major but preventable public health problem associated with unfavorable pregnancy outcomes and developmental failures following birth [23]. In spite of an improvement in the implementation of universal salt iodization in Ethiopia, prenatal ID has not shown a significant reduction [24]. In this study, prenatal ID was 60.5% (95% CI 55.6, 65.5). It remains a major public health problem, which requires comprehensive nutritional strategies to mitigate the burden.

Furthermore, the finding of the study is far greater than those of studies conducted in Nepal (28.9%) [25], and Kolkata (37%) [26, 27]. Poor dietary intake of iodine-rich food and low utilization of iodized salt could explain the observed discrepancies. As an illustration, iodized salt utilization coverage in Nepal was (66.7%) [28], which is far better than Ethiopia (23.3%) [17].

Though our finding has shown a significant low prevalence of ID compared to previous studies conducted in Ethiopia (88.9%), and (82.8%) [7, 29], the burden is still unacceptably high. This might be related to improvements in the utilization of iodized salt and public awareness as a result of aggressive media promotion regarding the importance of iodine. Furthermore, in the former studies, only 6.6% of the households utilized iodized salt in 2013 [29]. However, this finding is in line with a study conducted in Gayint, and Niger [6, 20], which amounted to be 61.4%, and 61.6%, respectively.

Women whose husbands were government employees were found with 60% lesser odds of developing ID than women whose husbands were unemployed. Obviously, unemployment is a proxy indicator of lower-household income which adversely affects the food security. A study in China also showed that low-income was significantly associated with the development of micronutrient deficiency [30, 31]. Likewise, the current and previous local findings re-affirmed the negative effect of poor-household income on the risk of ID [7].

This study also revealed that the odds of ID were higher among women in the second trimester of pregnancy than the third trimester. This finding is in agreement with an earlier study in Ethiopia [20]. Similarly, the finding is supported by a study in the UK [32], which reported a better concentration of urine iodine in the third than first and second trimester. This is because firstly, fetal thyroid gland formation begins at 12 weeks and ends in the second trimester. Secondly, at the 20th week of gestation, the fetal thyroid gland starts to synthesize the thyroid hormone with the assistance of a maternal hormone [33]. As a result, lesser iodine is required. Moreover, the demand for Human Chorion Gonadotropins (HCG) production during the third trimester is not much required [34].

Although cabbage consumption has many health benefits, its overconsumption leads to bad consequences. The current study explored that cabbage consumption twice and more per week would result in ID. This finding is similar to studies conducted in Ethiopia [7, 29]. The substance thiocyanates found in cabbage and other cruciferous vegetables compete with the uptake of iodine by thyroidal cells; consequently, the activity of the Thyroid Peroxidase (TPO) enzyme is impaired [35]. This explanation is further complemented by another study done in Bulgaria which showed women who had low urinary iodine were found to have high thiocyanates concentration in their urine and TSH [36]. Even though literature has not shown enough on the effect of maize on ID among the human species, this study revealed that maize consumption is positively associated with the ID. A study in an animal model supports this finding. This study pointed out that a substance (thiocyanate) found in maize caused ID by interfering with the activities of TPO [37].

All in all, ID among pregnant women was high which depicts a mild public health problem. Maize consumption, cabbage consumption (twice or more times in a week), being in the second trimester of pregnancy, and poor household wealth status have increased the likelihood of ID. Therefore, special attention for women during the second trimester of pregnancy, enhancing of household income, and nutrition education to minimize maize, as well as cabbage consumption are recommended.

## Limitations

The study is not believed to be free from some limitations. Firstly, it has not determined household salt utilization status. Secondly, recall and social desirability bias regarding dietary practices could not be ruled out.

## Abbreviations

ANC: antenatal care
EDHS: Ethiopian Demographic Health Services
G/dl: gram per deciliter
ID: iodine deficiency
IQ: Intelligent Quotient
ML: milliliter
MoH: Ministry of Health
MUAC: Mid Upper Arm Circumference
TSH: thyroid stimulating hormone
μ/L: microliter
MUIC: Median Urinary Iodine Concentration
